# Nanoforming Hyaluronan-Based Thermoresponsive Hydrogels: Optimized and Tunable Functionality in Osteoarthritis Management

**DOI:** 10.3390/pharmaceutics14030659

**Published:** 2022-03-17

**Authors:** Alexandre Porcello, Paula Gonzalez-Fernandez, Olivier Jordan, Eric Allémann

**Affiliations:** 1 School of Pharmaceutical Sciences, University of Geneva, CH-1206 Geneva, Switzerland; alexandre.porcello@unige.ch (A.P.); paula.gonzalezfernandez@unige.ch (P.G.-F.); olivier.jordan@unige.ch (O.J.); 2 Institute of Pharmaceutical Sciences of Western Switzerland, University of Geneva, CH-1206 Geneva, Switzerland

**Keywords:** hyaluronic acid, biodegradation, rheology, thermoresponsiveness, viscosupplementation

## Abstract

Hyaluronic acid (HA) constitutes a versatile chemical framework for the development of osteoarthritis pain treatment by means of injection in the joints, so-called viscosupplementation. Without appropriate physico-chemical tuning, such preparations are inherently hindered by prompt in vivo degradation, mediated by hyaluronidases and oxidative stress. To prolong hydrogel residence time and confer optimized product functionality, novel thermoresponsive nanoforming HA derivatives were proposed and characterized. Combined use of sulfo-dibenzocyclooctyne-PEG4-amine linkers and poly(*N*-isopropylacrylamide) in green chemistry process enabled the synthesis of HA-based polymers, with in situ obtention of appropriate viscoelastic properties. Spontaneous and reversible thermoformation of nanoparticles above 30 °C was experimentally confirmed. Lead formulations were compared to a commercially available HA-based product and shown significantly better in vitro resistance to enzymatic and oxidative degradation, required half the injection force with optimal viscoelastic hydrogel properties in equine synovial fluids. Results highlighted the vast potential of appropriately engineered HA-based systems as next-generation long-acting viscosupplementation products for osteoarthritic patients.

## 1. Introduction

Viscosupplementation constitutes an important therapeutic approach for knee osteoarthritis (OA), exogenously and artificially supplementing synovial fluid HA content through intra-articular injection, thereby promoting joint lubrication recovery. As a preponderant constituent of synovial fluid (i.e., composition and function), a natural decrease of HA contents or shifts in HA molecular weight (MW) with physiological aging is often linked with the onset and progression of OA [1,2]. HA is a natural linear polyanionic polymer composed of alternating units of glucuronic acid and N-acetylglucosamine, connected by β-linkages, weighing up to 11 MDa in healthy human synovial fluid [1,2,3,4,5,6]. Physiological HA presents unique rheological properties and plays versatile significant roles as a lubricant, shock absorber, and matrix in the joint [5,7]. Inflammation, aging, hyaluronidases activity, and oxidative stress appear to contribute to HA degradation and OA progression [3,4].

Therapeutic management of knee OA is often undertaken using high molecular weight (HMW) HA intra-articular injection products [7,8,9]. However, most available reviews on HA-based OA viscosupplement products mention a lack of homogeneity among clinical evaluations, mitigating the conclusions to be drawn on the effectiveness of such therapies [10,11,12]. The observed suboptimal clinical responses may be due to insufficient retention of biopolymers in the joint, caused by rapid enzymatic degradation and oxidative stress, mediated by reactive oxygen species (ROS) [13,14,15]. Actually, HA carboxylate groups are known to be recognized by HYAL-2 enzymes, and both HA structural component units are susceptible to hydroxyl radical attacks (e.g., scission of the glycosidic bond) [16,17,18,19]. Considering the in vivo environment of product application, ROS are involved in the mechanisms of degradation of HA, in chondrocyte apoptosis, as well as production and activation of collagenase matrix metalloproteinase-1 [15,16,17,18,19]. Despite the ongoing debate around the clinical efficacy of viscosupplementation, HA injections remain a common practice among rheumatologists.

Due to the aforementioned factors potentially hindering the efficacy of HA-based injectable OA formulations, several chemical derivatization strategies (e.g., crosslinking, chemical functionalization, combinations) have been investigated to reduce multifactorial degradation and maximize hydrogel residence time [15,19,20,21,22,23,24]. Specific HA conjugates comprising the thermoresponsive polymer poly(*N*-isopropylacrylamide) (PNIPAM) and a linker were recently proposed [20,25]. When PNIPAM is specifically linked to HA via a cyclooctyne linker and a PEG spacer, the copolymer spontaneously forms submicron spherical particles above a defined lower critical solution temperature (LCST). Coined “HA-L-PNIPAM” or “HA Nano”, such formulations were observed to form nanoparticles upon injection in mice and provided extended residence time attributed to reduced enzymatic degradation, potentially improving therapeutic effects in the joint [20].

Based on the previous promising proof of concept, we aimed to design a new thermoresponsive and optimal injectable HA-based viscosupplement formulation for OA management. The choice of the linker in addition with our green chemistry synthetic route facilitates the polymer aqueous dissolution. The news rheological properties of the formulations at body temperature were developed to circumvent the shortcomings of commercial HA. The formulation was further compared to a reference product (i.e., Ostenil^®^, TRB Chemedica, Geneva, Switzerland), in terms of viscoelastic properties (at a shear rate simulating walking and running), injectability, and in vitro resistance to hyaluronidase and ROS-mediated degradation. Apart from OA applications, HA-L-PNIPAM could potentially serve as drug-delivery vehicle and in other biomedical applications requiring highly versatile polymers.

## 2. Materials and Methods

### 2.1. Materials

*N*-(3-dimethylaminopropyl)-*N*′-ethylcarbodiimide (EDC), *N*-hydroxysuccinimide (NHS), azide-terminated poly(*N*-isopropylacrylamide) (PNIPAM-N_3_; 15 kDa), amine-terminated poly(*N*-isopropylacrylamide) (PNIPAM-NH_2_; 5.5 kDa), hyaluronidase from bovine testes (Type VI-S), hydrogen peroxide (30% *w/w*) and all medium for cell culture were purchased from Sigma-Aldrich™, St. Louis, MO, USA. Laboratory grade hyaluronic acid (HA) sodium salt (2200–2400 kDa) was purchased from Contipro, Dolní Dobrouč, Czech Republic. Sulfo-Dibenzocyclooctyne-PEG4-amine (Sulfo DBCO-PEG4-NH_2_) was bought from Broadpharm, San Diego, CA, USA. Dialysis membranes (Biotech CE Dialysis Tubing 50 kDa) were acquired from Repligen, Waltham, MA, USA.

### 2.2. Synthesis of HA-L-PNIPAM 0.5, HA-L-PNIPAM 0.25 and HA-L-PNIPAM 0.1

Various HA-L-PNIPAM polymers were synthesized using a modified procedure [20]. Practically, HA (2200–2400 kDa) was dissolved in distilled water at 0.2% *w/v* under magnetic stirring. After dissolution of the polymer, EDC (10 eq. COO^−^) and NHS (3 eq. COO^−^) were added at respective intervals of 15 min. The pH was adjusted to 5.5, using 0.1 M NaOH and 0.1 M HCl. Then, Sulfo DBCO-PEG4-NH_2_, previously dissolved in distilled water, was added (0.5 eq. COO^−^ for HA-L-PNIPAM 0.5, 0.25 eq. COO^−^ for HA-L-PNIPAM 0.25 and 0.1 eq. COO^−^ for HA-L-PNIPAM 0.1). The reaction was allowed to proceed for 12 h under stirring at room temperature. The reaction mixture (HA Sulfo DBCO-PEG4) was dialyzed three times against 5% (*w/v*) NaCl (50,000 MWCO, 4 h, room temperature) and three times against distilled water (50,000 MWCO, 4 h, at room temperature) before being transferred to a round bottom flask. PNIPAM-N_3_ (1 eq. DBCO) was added under stirring for 6 h, and the pH was adjusted to 7, using 0.1 M NaOH. The product was dialyzed three times against 5% (*w/v*) NaCl (50,000 MWCO, 4 h, 4 °C) and six times against distilled water (50,000 MWCO, 2 h, 4 °C), before being frozen at −80 °C, lyophilized (Freeze Dryer Alpha 1–4 LDplus, Christ, Osterode am Harz, Germany; 48 h, 1.5 × 10^−1^ mbar, −80 °C) and stored at 4 °C. Chemical structures and degrees of substitution (DS) of the intermediate HA-Sulfo DBCO-PEG4 (DS_1_) and of the final product (DS_2_) were assessed by ^1^H NMR spectroscopy after hyaluronidase enzymatic digestion on a Bruker Avance Neo 600 MHz NMR spectrometer using D_2_O as a solvent. The DS were determined by comparing the integration of methyl protons of HA with the aromatic protons of DBCO for the intermediate HA-Sulfo DBCO-PEG4 (DS_1_) and the integration of the methyl protons of PNIPAM with the aromatic protons of DBCO for the final product (DS_2_). DS_1_ were confirmed by UV-vis spectroscopy, and reactions were followed by Fourier transform infrared (FT-IR) spectrometry.

### 2.3. Synthesis of HA-PNIPAM

As a control polymer, PNIPAM was directly coupled to HA without DBCO and spacer. HA was dissolved in distilled water at 0.2% *w*/*v* under magnetic stirring. After the dissolution of the polymer, EDC (10 eq. COO^−^) and NHS (3 eq. COO^−^) were added at respective intervals of 15 min. The pH was adjusted to 5.5. Then, PNIPAM-NH_2_ dissolved in distilled water was added (0.5 eq. COO^−^). The reaction was allowed to proceed for 12 h under stirring at room temperature. HA-PNIPAM was dialyzed three times against 5% *w/v* NaCl (50,000 MWCO, 4 h, 4 °C) and six times against distilled water (50,000 MWCO, 2 h, 4 °C), before being lyophilized and stored at 4 °C. DS of HA-PNIPAM was determined by UV-vis spectroscopy and gravimetry. Reaction was followed by Fourier transform infrared (FT-IR) spectrometry.

### 2.4. Size and Morphology of the Self-Assembled Nanoparticles

The mean size and the formation process of nanoparticles were assessed continuously using dynamic light scattering (DLS) (Nanosizer, Malvern, UK), on temperature steps (i.e., 0.5 °C/step, 10 min step duration) ranging from 22 °C to 37 °C. HA-L-PNIPAM 0.5, HA-L-PNIPAM 0.1, and HA-PNIPAM were formulated at three concentrations (0.1%, 0.01%, and 0.1% *w*/*v*) in PBS buffer. The morphology of nanoparticles was observed using scanning electron microscopy (SEM) (JEOL microscope, JSM-7001TA, Tokyo, Japan). The samples were prepared by vacuum drying a drop of formulation (0.001% *w*/*v*) heated at 37 °C on carbon tape. The SEM samples were sputter-coated with a 20 nm gold layer (Leica EM SCD 500, Leica Microsystems GmbH, Wetzlar, Germany), then observed with a scanning electron microscope at 15 kV.

### 2.5. In Vitro and Ex Vivo Rheological Behavior

Rheological behaviors of HA-L-PNIPAM at different concentrations (% *w*/*v*) in PBS buffer and Ostenil^®^ were determined on a HAAKE Mars Rheometer™ (Thermo Scientific, Waltham, MA, USA) equipped with a Peltier cone-plate C35 2°/Ti measuring geometry on 420 µL samples. The storage modulus (G′) and loss modulus (G″) were assessed as a function of temperature, using a ramp from 22 °C to 37 °C with a heating rate of 0.04 °C/s and a constant oscillatory frequency of 0.5 Hz and 2.5 Hz, simulating walking and running conditions, respectively [26]. A sample hood was used during the measurements to minimize evaporation. Shear stress was set to 0.5 N/m^2^ in all experiments to respect the linear viscoelastic region (LVE). Rheological behaviors of HA-L-PNIPAM 0.5 and Ostenil^®^ mixed with equal volumes of equine synovial fluid were determined at 22 °C and 37 °C at 0.5 and 2.5 Hz. Equine synovial fluid was aspirated from six fetlock joints, obtained after slaughter from three adult horses. Aspirated synovial fluid was kept in a refrigerator at approximately 4 °C, for testing within 48 h after collection.

### 2.6. Injectability of Formulations

The force injection profile of a syringe filled with different concentrations of HA-L-PNIPAM 0.5 and with Ostenil^®^ were determined at 22 °C using a speed of 2 mm·s^−1^ on a Texture Analyzer TA.XT. Plus (Tracomme AG, Schlieren, Switzerland). Syringes (BD with Luer-Loc™ Tip) fitted with a 23G needle (i.e., generally used for clinical injections in joints) were used.

### 2.7. Enzymatic Degradation Assays

Accelerated enzymatic degradation of the polymers was dynamically assessed by means of rheology. A volume of 40 µL of hyaluronidase (100 U/mL) was added to 400 µL of the sample before a 30 s homogenization and rheological measurement. The storage modulus (G′) and loss modulus (G″) were measured as a function of time (i.e., over 12 min) at a constant oscillatory frequency of 0.5 Hz. The delay between the addition of hyaluronidase and the first measurement was two minutes. As a control condition, 40 µL of PBS buffer were added to 400 µL of the sample, ratio was made at each timepoint. Long-term degradation assays were performed using lower concentrations of the enzyme by sequential addition of 10 µL volumes of hyaluronidase (i.e., 50 U/mL added on days 0, 7, 14, 21) in 400 µL of the samples, which were incubated at 37 °C under gentle shaking. After 28 days, G′ and G″ were measured at a constant oscillatory frequency of 0.5 Hz.

### 2.8. Degradation by Induction of Oxidative Stress

To generate oxidative stress, 40 µL of H_2_O_2_ (30% *w/w*) were added to 400 µL of sample. H_2_O_2_-mediated degradation was assessed by rheology, using the same method as described above. Long-term degradation assays were performed by the addition of 40 µL of H_2_O_2_ (30% *w/w*) to 400 µL of sample, which was incubated at 37 °C under gentle shaking. After six hours, G′ and G″ were measured at a constant oscillatory frequency of 0.5 Hz.

### 2.9. Product Sterilization

HA-L-PNIPAM sterilization was performed using Stericup^®^ vacuum filtration systems with 0.22 µm pore sizes (Millipore^®^, Merck, Darmstadt, Germany). HA-L-PNIPAM 0.5 were diluted in milli-Q^®^ water at a concentration of 0.05% *w/v* before filtration. Formulations were then lyophilized before storage at 4 °C. The viscosity of resuspended sterile HA-L-PNIPAM 0.5 was measured at constant shear stress (0.1 s^−1^), to evaluate the effect of the sterilization process.

### 2.10. In Vitro Cytotoxicity Assay on Human Fibroblast-like Synoviocytes

Isolation and culture of Human Fibroblast-like Synoviocytes (HFLS) was performed as previously described [27]. Hip synovial membrane was collected from three male adult patients with clinical OA at the time of hip replacement surgery. This protocol was conducted under the approval of the local Ethics Committee (CCER, Geneva, Switzerland) (Authorization #2017-02234) and with informed and consenting patients. Briefly, once collected the samples were finely minced and digested for 3 h (37 °C, 5% CO_2_ incubation) in a 3 mg/mL collagenase IX-RPMI 1640 solution. After centrifugation (200× *g*) and supernatant removal, the resuspended pellet was cultured (37 °C, 5% CO_2_) in medium containing an equal ratio of RPMI 1640 and M199 with 1% penicillin/streptomycin (100 IU/mL:100 g/mL), 2 mM l-glutamine and 20% fetal bovine serum. Nonadherent cells were removed after 12 h. After four passages confluent HFLS cells (25,000 cells/well) were treated in a 96-well plate with several formulations and compounds (i.e., HA-L-PNIPAM 0.5 before and after hyaluronidase enzymatic digestion, PNIPAM-N_3_, Sulfo DBCO-PEG4-NH_2_, Ostenil^®^, and HA 2200–2400 kDa) at different concentrations for 24 h. PBS was used as solvent for all conditions. After the 24 h and 72 h of incubation time adherent cells were tested for viability using the cell proliferation reagent WST-1 (Abcam, Cambridge, England), according to the supplier instructions.

### 2.11. Statistical Analysis

Data are expressed as mean ± standard deviation (s.d.). For statistical significance of the datasets from experiments, a one-way ANOVA test was performed, and was followed by a post hoc Tukey’s multiple comparison test. A *p* value < 0.05 was retained as a general base for statistical significance determination. Detailed levels of statistical significance were detailed further in the Results section. Statistical calculations and/or data presentation were performed using GraphPad Prism v. 8.0.2 (GraphPad Software, San Diego, CA, USA).

## 3. Results and Discussion

### 3.1. Synthesis and Characterization of the HA Derivatives

**Figure 1:**
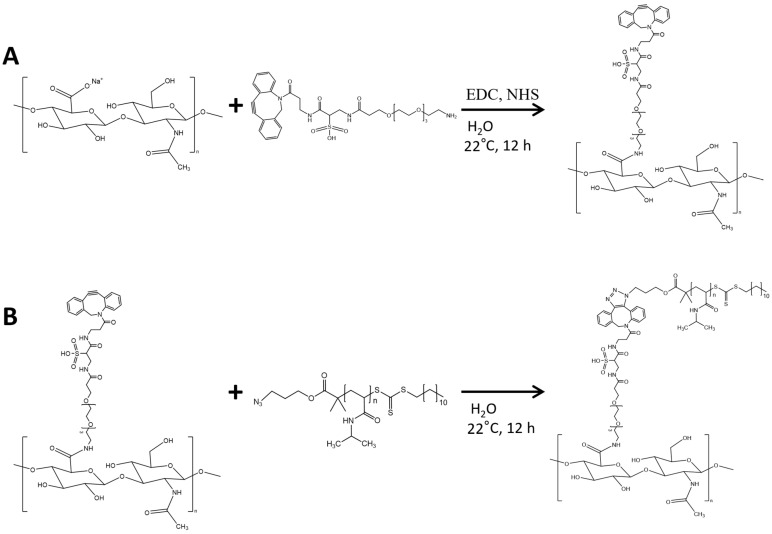
Synthetic route of HA-L-PNIPAM. Amidation reaction (**A**) and copper-free azide-DBCO click chemistry reaction (**B**).

The green chemistry synthetic route for HA-L-PNIPAM formulations is presented in Figure 1. There are several methods and conditions to conjugate amine groups to the carboxylic acid of the HA backbone. To use only water as solvent, EDC and NHS reagents were used in a slightly acidic environment (i.e., pH = 5.5) to activate and stabilize the HA carboxylic acid group [28]. Briefly, HA (2200–2400 kDa) was derivatized in an amidation reaction with a water-soluble linker, Sulfo DBCO-PEG4-NH_2_, in the presence of a large excess of EDC and NHS. Then, the DBCO group of the linker reacted via copper-free azide-DBCO click chemistry with PNIPAM-N_3_ (15 kDa, 1 eq. DBCO). The final yield was 70%. The DS was calculated from the ^1^H NMR spectrum. The aromatic protons of DBCO allowed the calculation of the DS_1_ of the amidation from the NMR spectra, with values up to 16.9% (Figure S1) [20,29]. The DS_2_ for the azide-DBCO grafting with the CH_3_ groups of PNIPAM units was of 86.3% (Figure S1). These results were in accordance with the literature, indicating that amidation of HA with EDC and NHS in water led to relatively low DS values, whereas azide-DBCO click chemistry led to a very high substitution [29,30]. Using the same synthetic route, the DS_1_ for HA-L-PNIPAM 0.25 is 6.25% for the amidation, and 87.5% for the azide-DBCO click chemistry (Figure S2), the DS_1_ for HA-L-PNIPAM 0.1 is 1.125% for the amidation, and 49.5% for the azide-DBCO click chemistry (Figure S3). Results of similar value ranges were obtained by UV-vis spectroscopy for DS_1_ (Table S1). ^1^H NMR spectrum of HA-PNIPAM is presented in Figure S4. ATR/FT-IR (Figure S5) provides further evidence for the formation of HA-L-PNIPAM, with the characteristic absorption peaks of PNIPAM at 3284 cm^−1^ (N–H stretching) and compared to HA spectrum the new signals of the conjugates at 1638 and 1535 cm^−1^ (amide bands) suggested the formation of a new amide bond. Serving as controls and comparative formulation, HA-PNIPAM was synthesized without the addition of a linker, through the same amidation reaction using amine-terminated PNIPAM (5.5 kDa, 0.5 eq.). According to the UV spectrum and the final weight the DS for HA-PNIPAM was of 8% (Table S2). ATR/FT-IR (Figure S6) provides further evidence for formation of HA-PNIPAM with characteristic absorption peaks of PNIPAM and amide.

**Figure 2:**
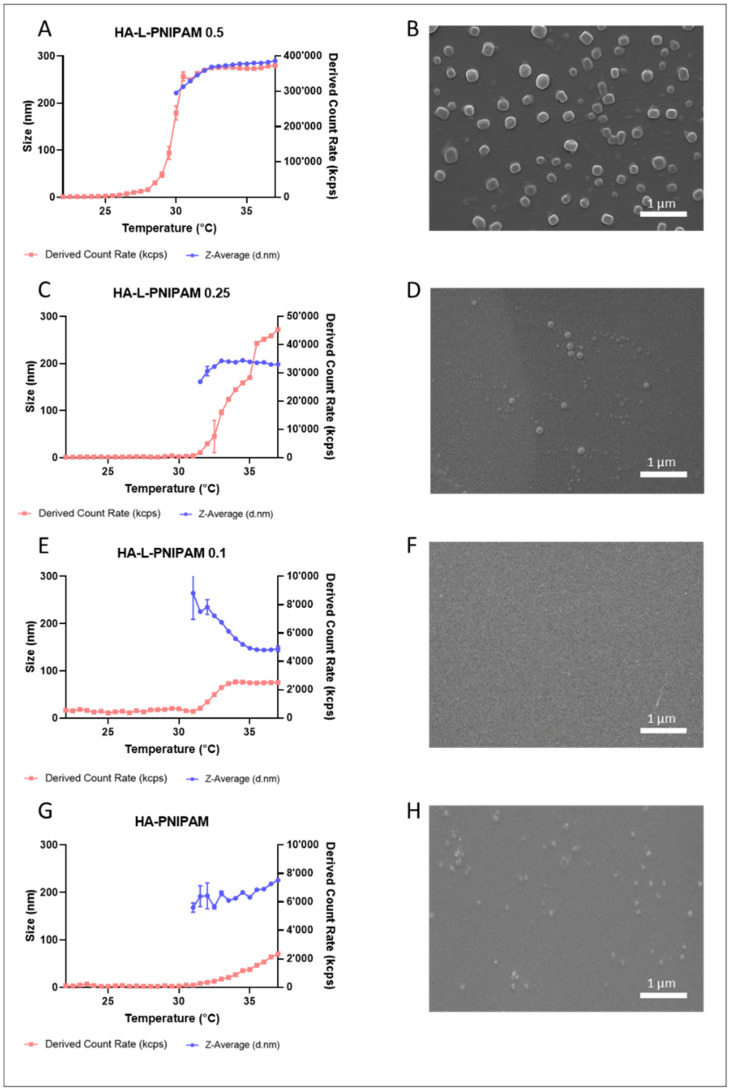
Formation process of nanoparticles upon heating for HA-L-PNIPAM 0.5 (PDI from 0.273 to 0.011) (**A**), HA-L-PNIPAM 0.25 (PDI from 0.464 to 0.212) (**C**), HA-L-PNIPAM 0.1 (PDI from 0.265 to 0.169) (**E**) and HA-PNIPAM (PDI from 0.276 to 0.008) (**G**). Their size and the derived count rate are presented as a function of temperature (*n* = 3; ±sd). Scanning electron micrograph of HA-L-PNIPAM 0.5 (**B**), HA-L-PNIPAM 0.25 (**D**), HA-L-PNIPAM 0.1 (**F**) and HA-PNIPAM (**H**) of samples. Scale bar = 1 µm. Magnification 20,000×. Voltage = 15.0 kV. Concentrations 0.001% *w/v*.

In this work, HA-based hydrogels were formulated by mixing the lyophilized polymers with appropriate volumes of PBS buffer. Compared to previous work, the use of the hydrophilic sulfonated linker was a major improvement to increase the water solubility of the DBCO function [20]. Due to the well-described thermosensitive properties of PNIPAM, the polymeric architecture of its grafting with DBCO, and the high water-solubility of final HA-L-PNIPAM formulations, the synthesized polymers displayed unique temperature-dependent properties as well as spontaneous and reversible formation of nanostructures above the LCST (Figure 2A,C,E) [20,25,31]. Ostenil^®^ and native HA controls did not form any nanostructures at comparable temperatures (data not shown). With HA-PNIPAM a very limited number of nanostructures formed above the defined LCST. As evidenced by DLS (Figure 2G) and SEM (Figure 2H), in contrast with HA-L-PNIPAM 0.5 formulations (Figure 2A,B), a significantly lower concentration of nanostructures was observed for HA-PNIPAM. Above the LCST, HA-L-PNIPAM 0.1 nanoparticles (Figure 2C) had a significantly smaller size (i.e., around 140 nm) than with HA-L-PNIPAM 0.25 and HA-L-PNIPAM 0.5 and a reduced concentration of nanostructures. Higher DS values (i.e., higher PNIPAM content) correlated with a relatively higher particle concentration and larger sizes. For an appropriate comparison, all samples were diluted for SEM analysis (0.001% *w*/*v*), revealing the spherical morphology of the nanoparticles formed within HA-L-PNIPAM 0.5. Analysis of HA-L-PNIPAM 0.25, HA-L-PNIPAM 0.1 and HA-PNIPAM samples (Figure 2D,F,H) showed very low concentrations of nanoparticles. Z-averages were not measurable between 22 °C to 30 °C for HA-L-PNIPAM 0.5, and 22 °C to 31 °C for all the other formulations, due to the lack of particles at these temperatures (too low count rate values are attributed to the absence of nanostructure formations). Higher derived count rates usually indicate higher concentration values or larger particle sizes. During data acquisition with a temperature ramp, the size of the particles measured rapidly increased to reach a plateau at 290 nm, 200 nm and 140 nm for HA-L-PNIPAM 0.5, HA-L-PNIPAM 0.25 and HA-L-PNIPAM 0.1, respectively. A plateau was not reached for HA-PNIPAM at 37 °C, the particles formed had a mean size close to 220 nm. These results confirmed that the type of linker plays a major role in shaping both size and concentration of the nanoparticles, as well as the DS. Formulations with DBCO comprising more grafted PNIPAM lead to higher nanoparticle concentrations and larger sizes. At temperatures below the defined LCST, no particles were present, confirming the single “sol” state of the product. Above the LCST, particles were clearly present. This sudden specific thermoresponsive nanoparticle formation occurred between 27 to 30 °C for HA-L-PNIPAM 0.5. It happened at a higher temperature for HA-PNIPAM (i.e., 31 °C). The differences in LCST values are mainly linked to the molecular weight of PNIPAM, as demonstrated in separate studies (data not shown). Higher derived count rates for HA-L-PNIPAM 0.5 may be due to presence of larger nanoparticles and higher concentrations thereof. Based on the DLS and SEM results, HA-L-PNIPAM 0.5 formulation was chosen for further analysis and comparison with the commercial product.

### 3.2. Rheology and Injectability

**Figure 3:**
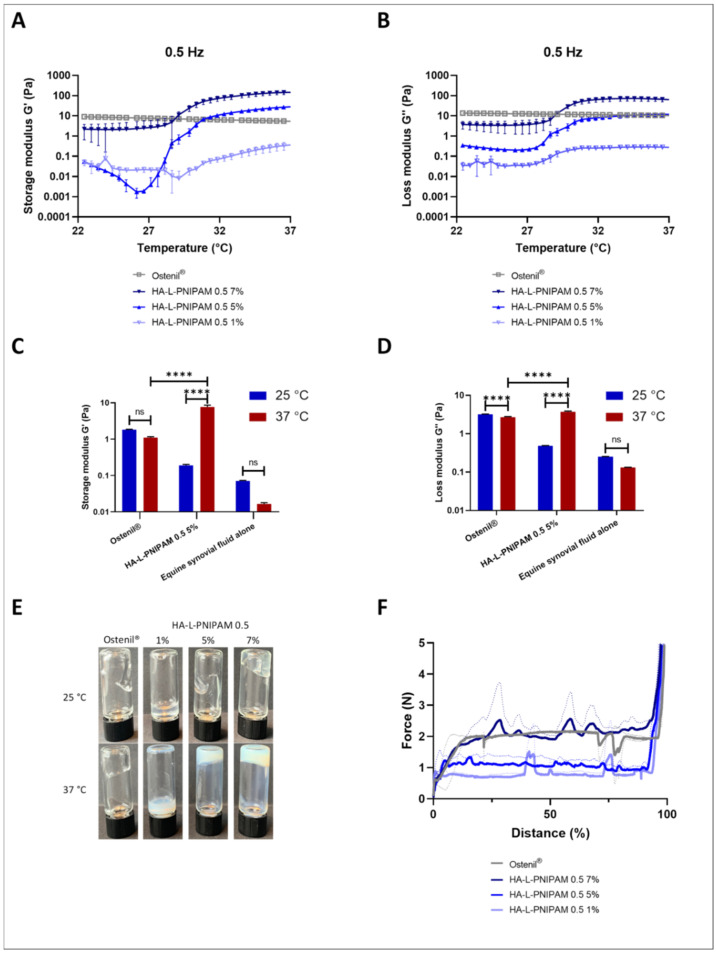
Temperature dependence of the storage modulus (G′) and loss modulus (G″) at a frequency of 0.5 Hz of Ostenil^®^, HA-L-PNIPAM 0.5 1%, 5%, and 7% (*n* = 3; ± sd) (**A**,**B**). Storage modulus G′ (**C**) and loss modulus G″ (**D**) of Ostenil^®^ and HA-L-PNIPAM 0.5 5% mixed with equal volumes of equine synovial fluid and equine synovial fluid alone at 22 °C and 37 °C at a frequency of 0.5 Hz. Highly statistically significative differences (i.e., *p* < 0.0001) were evidenced by four asterisks (“****”) (*n* = 3; ±sd) (**C**,**D**). Picture of Ostenil^®^, HA-L-PNIPAM 0.5 1%, 5%, and 7% at 25 °C and 37 °C after a 180 ° flip of the vial, and 15 s delay (**E**). Force in Newtons (N) required to extrude Ostenil^®^, HA-L-PNIPAM 0.5 1%, 5%, and 7% as a function of the stroke distance of the piston in a syringe with a 23G needle (distance %) at 22 °C (*n* = 2; ±sd) (**F**).

Viscoelastic properties of HA conjugates and the reference product were determined as a function of temperature at adequate oscillatory frequencies of 0.5 Hz (Figure 3A,B) and 2.5 Hz (Figure S7), simulating walking and running conditions, respectively [26]. Average data obtained from three different measurements, and the corresponding standard deviations at 25 °C and 37 °C, are reported in Table 1.

A well-defined increase in both loss and elastic moduli G″ and G′ corresponded to the LCST of HA-L-PNIPAM 0.5. The storage modulus G′, which reflects the elastic behavior of the material, displayed a transition, with a first slight reduction of values for HA-L-PNIPAM 1% at both frequencies and 5% at 0.5 Hz. This change in viscoelastic properties occurred between 27 °C to 30 °C at both frequencies, which could then be considered as the rheological LCST, as confirmed by the results obtained in DLS. These transitions arose from the thermosensitive behavior provided by PNIPAM grafting [20]. As the temperature increased above the LCST, a transition from a loss modulus prevalence (G″ > G′) to a gel-like state with a storage modulus prevalence regime (G′ > G″) was observed. This shift occurred within a limited temperature range of less than 4 °C. Thus, in practice, the low viscosity of HA-L-PNIPAM at room temperature would enable an easy injection of the formulation. Once injected, and since the body temperature generally exceeds the LCST of the HA conjugate, a spontaneous phase transition would occur, with a significant increase in product viscoelastic properties (i.e., from 19 to 2900-fold for G′ and from 9 to 54-fold for G″ depending on the concentration). Increasing the viscosity in the pathological joint space is correlated with the greater efficacy of HA-based products [7]. Measurements performed on Ostenil^®^ showed a continuous decrease of G′ and G″ as temperature increased from 22 to 37 °C. Viscosity declined with the temperature increase. This was congruent with theoretical predictions, as liquid compounds generally present lower viscosity values at increased temperatures. During all the analyses, G″ remained superior to G′, indicating that the sample remained mainly viscous in the temperature range of the study. No phase transition could be observed. Our results showed that below the LCST (i.e., at 25 °C), the viscoelastic properties G′ and G″ at all HA-L-PNIPAM 0.5 samples at all concentrations remained inferior to those of Ostenil^®^. Above the LCST, compared to the reference G′ and G″ of Ostenil^®^, HA-L-PNIPAM 0.5 showed values approximately 10-fold lower at 1% (*w/v*) concentration, in a comparable range for 5%, and values 5- to 29-fold higher for 7%. In all cases, the viscoelasticity values G′ and G″ were higher at the frequency corresponding to running conditions (2.5 Hz) (Figure S7 and Table S3). These results are in accordance with the lower overall HA content for HA-L-PNIPAM 0.5 formulations (Table 1), despite a reported HA MW in Ostenil^®^ of 1.6 MDa, comparatively lower than the HA used in HA-L-PNIPAM synthesis.

Considering the specificity and temperature-dependent characteristics of HA-L-PNIPAM formulations, products incorporating such HA-based polymers may be tailored by adjusting the HA MW, the DS, and the final concentration of the polymer, to obtain defined viscoelastic properties. HA-L-PNIPAM 0.5 at 5% (*w/v*) was chosen for further rheological analysis and compared to the commercial linear HA, since at 37 °C, both had similar viscoelastic properties.

The ability of HA-L-PNIPAM 0.5 5% to increase in viscosity above the LCST was experimentally confirmed in a relevant biological fluid (i.e., equine synovial fluid, Figure 3C,D). For these trials, the formulations were mixed with equal volumes of equine synovial fluid before rheological analysis at 22 °C and 37 °C. The robustness of the thermosensitive system was proven by the amplitude of both modulus changes upon temperature increase (i.e., at 25 °C and at 37 °C), despite the complexity of the medium. Results showed an increase of both moduli at 37°C (i.e., 40-fold for G′ and eightfold for G″) in the mixture with equine synovial fluid. In the same experimental conditions, at 37 °C, compared to the reference G′ and G″ of Ostenil^®^, HA-L-PNIPAM 0.5 showed higher viscoelastic properties (i.e., 6.9-fold for G′ and 1.3-fold for G″).

Photographs demonstrating the change of viscosity and appearance for HA-L-PNIPAM 0.5 and Ostenil^®^ at 25 °C and 27 °C are shown in Figure 3E. The pictures were taken exactly 15 s after flipping upside down the vials. The increase of viscosity at 37 °C was macroscopically evident for HA-L-PNIPAM 0.5 at 5% and 7%. The color and opacity variations increased with the concentration of HA-L-PNIPAM 0.5. The ease of injection constitutes a critical parameter for all types of HA injectable products (e.g., viscosupplementation, dermal fillers, and reconstructive surgery) and was characterized by the injection profile of HA-L-PNIPAM formulations. Three concentrations of HA-L-PNIPAM and Ostenil^®^ were evaluated with a syringe fitted with a 23G needle commonly used for clinical intra-articular injections (Figure 3F). In comparison to Ostenil^®^, HA-L-PNIPAM 0.5 1% and 5% displayed lower injection forces, close to three times less force for HA-L-PNIPAM 0.5 1% and two times less force for HA-L-PNIPAM 0.5 5% at the plateau. The injection profile of HA-L-PNIPAM 0.5 7% was measured as being in the same range than the reference product, although having a very high concentration of polymer.

Overall, HA-L-PNIPAM 0.5 conferred higher viscoelastic properties to the synovial fluid, with an ease at injection.

### 3.3. In Vitro Degradation, Sterilization and Cytotoxicity

**Figure 4:**
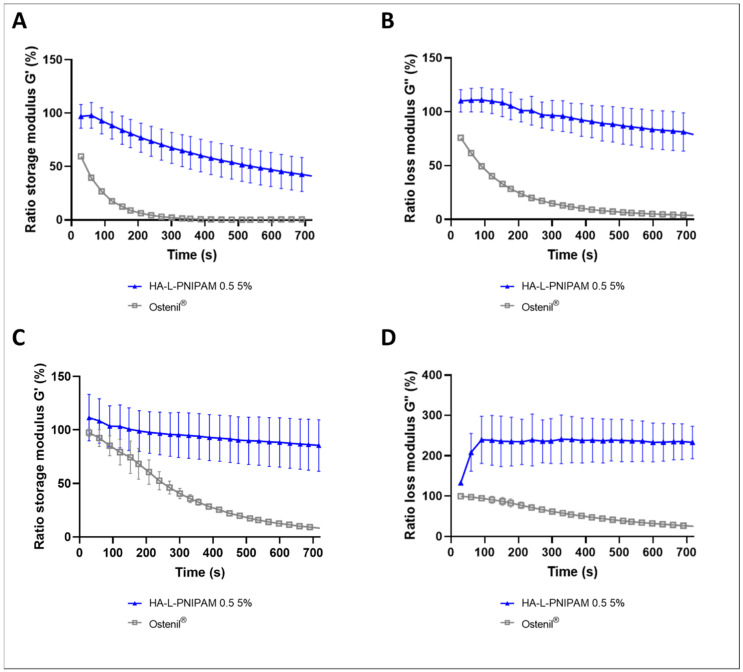
Ostenil^®^ and HA-L-PNIPAM 0.5 5% storage modulus (G′) and loss modulus (G″), normalized to initial values, as function of time at 0.5 Hz and 37 °C, after addition of 40 µL of hyaluronidase (100 U/mL) (**A**,**B**) (*n* = 3; ±sd). After an addition of 40 µL H_2_O_2_ (30% *w/w*) (**C**,**D**) (*n* = 3; ±sd). As a control condition, 40 µL of PBS buffer were added to 400 µL of the sample, ratio was made at each timepoint.

The HA-L-PNIPAM 0.5 5% formulation was compared to the reference product (i.e., Ostenil^®^ linear HA of 1.6 MDa at 10 mg/mL) concerning enzymatic digestion and degradation by induction of oxidative stress at 37 °C. For these trials, HA-L-PNIPAM 0.5 was formulated at 5% in order to have viscoelastic properties at 37 °C similar to those of Ostenil^®^. Firstly, in an accelerated test, high hyaluronidase concentration was added to the samples before the evaluation of the viscoelastic modulus over a period of 12 min, using a frequency of 0.5 Hz (Figure 4A,B).

Results showed a decrease of both moduli for the polymers tested. After 12 min, Ostenil^®^ displayed a massive reduction from the initial values of up to 95%, compared to HA-L-PNIPAM 0.5, which displayed only a reduction of 20% for the loss modulus and approximately 60% for the storage modulus over the measurement period. The conjugation of HA carboxyl groups with appropriate linker and side chains in order to form nanometric structures at body temperature is a key element to improve HA resistance to hyaluronidase. The reduction of carboxyl groups and the steric hindrance above the LCST are two key elements explaining the reduced degradation rate of the HA-L-PNIPAM formulation [19,20]. Similar experimental conditions were used to evaluate the oxidative degradation induced by the addition of H_2_O_2_ at a final concentration of 2.72% (*v/v*) in the test-item (Figure 4C,D). Ostenil^®^ was rapidly degraded by hydrogen peroxide, as the initial elastic modulus was close to 10% and viscous modulus close to 2% after 5 min. The resistance to degradation was better for HA-L-PNIPAM 0.5 5%, with results higher than 100% of the initial viscous modulus, a first increase in values for 90 s, before a plateau close to 240% of initial values. The elastic modulus displayed a slight decrease, with an average of 85% over 12 min, but assorted with high standard deviations in comparison with the values obtained for Ostenil^®^. Similar trends were obtained for long term degradation (Figure S8). The exact mechanism leading to the increase in G″ remains unclear but these results may be explained mainly by the conformation adopted by the nanostructured gel containing HA and PNIPAM, wherein spheres are entangled in the gel network, protecting the hydroxyl groups of HA from degradation by ROS. Overall, the important modulus decreases in this accelerated oxidative degradation assays is in all probability due to the artificial and high concentrations of the in vitro experiments, the same levels of oxidative stress will not be encountered in vivo. Furthermore, the main advantages of HA-L-PNIPAM formulation reside in the endpoint relative increase in both modulus, which constitute markers of hydrogel resistance to enzymatic and oxidative degradation.

Clinical application of HA-based viscosupplement requires demonstration of product sterility. Sterilization by 0.22 μm filtration represents a good option depending on the partial loss of viscosity, MW, structural integrity, and function of the polymeric networks. HA-L-PNIPAM 0.5 sterilizing filtration (i.e., 0.22 μm) induced acceptable changes on viscosity values in rotational rheology (0.1 s^−1^) at 37 °C (i.e., a reduction of 25.5% as mean value) (Figure S9). Alternative and specific sterilization processes for HA-based derivative and medical devices are described in different normative documents (e.g., ISO 10993-7) [32].

**Figure 5:**
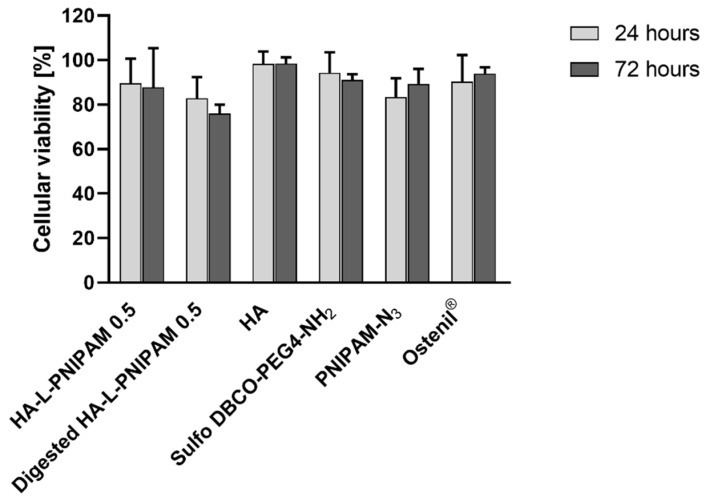
The cellular viability (WST-1 mitochondrial activity assay) of human fibroblast-like synoviocytes (HFLS) incubated with HA-L-PNIPAM 0.5 5%, HA-L-PNIPAM 0.5 5% digested by hyaluronidase, hyaluronic acid 0.71% (2200–2400 kDa), Sulfo DBCO-PEG4-NH_2_ 0.21%, PNIPAM-N_3_ 4.08% (15,000 Da) and Ostenil^®^ after 24 h and 72 h. No statistically significant differences (*p* > 0.05) with the control (cell culture medium) were observed for all conditions (*n* = 4; ±sd).

The cytotoxicity of HA-L-PNIPAM 0.5 5% and its components (i.e., HA, sulfo DBCO-PEG4-NH_2_ and PNIPAM-N_3_) at the same concentration than in the parent HA-L-PNIPAM 0.5 5% (Table 2) was assessed in comparison with the product of reference. Results are shown in Figure 5.

HFLS were assessed as viable after 24 and 72 h of incubation with the products and formulations tested, and no statistically significant differences of cellular viability levels were observed (Figure 5). These results were expected since copper-free click chemistry using DBCO is widely used and nontoxic. Comprehensive toxicity information is available [33]. Moreover, click chemistry precursors are already in clinical studies (e.g., NCT04106492) [34]. PNIPAM cytotoxicity tested at various molecular weights and concentrations is documented in the literature. The absence of cytotoxicity was confirmed [25,35]. HA-L-PNIPAM 0.5 at 5% was submitted to hyaluronidase degradation and was subsequently tested to mimic the in vivo degradation of the formulation, and to study the cytotoxicity of degradation products. Additional assays were performed on adult human chondrocytes and showed comparable results regarding the cytotoxicity parameters for HA-L-PNIPAM 0.5 5% (data not shown). Similar assays were performed on HA-L-PNIPAM 0.5 1% and 7% (Figure S10). Aspects of cells, results of viability obtained after trypan blue staining, and viscoelastic proper-ties after storage are shown in Figure S11, Table S4 and Figure S12. Overall, the HA-L-PNIPAM 0.5 formulation at 5% and its enzymatic degradation products showed no significant cellular toxicity.

### 3.4. HA-L-PNIPAM Design Consideration for Future Applications

Due to high physicochemical tunability and versatility, the described HA-L-PNIPAM polymers may be used as novel injectable biomaterials in human medicine, including for tendinous tissues disorders, volumetric supplementation of soft tissues, therapeutic cell or drug delivery systems, stress urinary incontinence, or in the field of aesthetic medicine as dermal fillers for example (Figure S13). Specifications for the appropriate and tailored design of HA-L-PNIPAM polymers depend on the intended application and clinical use. Depending on the eventual application, the targeted product profile may be very different. An optimal viscosupplement to treat OA should have a long residence time (i.e., high resistance to ROS and hyaluronidases), with an HMW HA to provide shock absrobtion and lubrication in the joint [8]. A dermal filler (e.g., labial volumizer) should have a high G′ value (i.e., >100 Pa) and a high cohesivity, and ultimately be injectable through very small, pain-free needles for an easier clinical use [36]. As for cell therapy, the key combination parameters for the clinical delivery of viable therapeutic cells must leverage a balance between a sufficient hydrogel viscosity, in order to maintain cells in suspension, and the reduction of the shear force applied on the cells during needle extrusion. In this specific context, the thermosensitive behavior of HA-L-PNIPAM would favor this balance, extending application to the injection of more sensitive cells and/or increasing cell survival, a criticial limitation of many actual cell therapies [37].

Generally, from a technical point of view during product development for a given application, HA-L-PNIPAM properties may be tailored by the choice of HA (i.e., MW, quality grade, polydispersity), where higher MWs will result in higher viscosity values of the reconstituted hydrogel system. As shown in the present study, the DS may be adapted during the polymer synthesis steps, leading to the incorporation of variable PNIPAM quantities. Therefore, additional experiments shall be conducted to further highlight all of the structure-function relationships linked to the DS. The final polymer concentration has an impact on the viscosity of the final formula at 25 °C and at 37 °C, respectively. Finally, HA-L-PNIPAM may be mixed with linear or crosslinked HA and may be used as an additive instead of the principal polymeric entity within the considered hydrogel system. Overall, it is possible to assess that many and various tools are available to tune HA-L-PNIPAM-based polymers and to use them for several therapeutic applications, and promising results were already obtained in a previous work performed in our laboratory using such hydrogels as a drug delivery system, despite low viscoelastic properties (i.e., G′ and G″ values < 8 Pa at 37 °C) [20]. As regards the delivery of viable therapeutic cells, proofs-of-concepts have already been established and reported for human progenitor tenocytes, with facilitated combination product injection and significant postinjection cell viability enhancements in products designed for musculoskeletal disorder (e.g., tendinopathies) management [38,39]. Overall, HA-L-PNIPAM could reach very high viscoelastic properties at body temperatures with no chemical crosslinkers while remaining injectable at room temperature with low viscoelastic properties.

## 4. Conclusions

In the present study, novel water-soluble HA derivatives based on an appropriate PNIPAM grafting, via a cyclooctyne linker and a PEG spacer, using green chemistry processes were synthetized, characterized, and compared to a commercial viscosupplementation product used to treat knee OA. The syntheses were controlled and resulting products may be sterilized by filtration. Rheological behavior, size and morphological measurements were performed, confirming spontaneous formation of spherical nanostructures (i.e., 290 nm in diameter) above a defined LCST. The significant role of the linker for spontaneous nanostructure formation in appropriate concentrations and related temperature-dependent properties was outlined, leading to adapted and high viscoelastic properties in PBS and equine synovial fluid. The potential uses and major benefits of HA-L-PNIPAM formulations are based on the unique thermoreversible, self-assembling properties of the derived HA polymer, enabling facilitated injection at room temperature. High viscoelastic values (i.e., comparable or higher than a commercial reference) are acquired in situ at body temperature, along with enhanced resistance to enzymatic and ROS-mediated degradation. The in vitro results presented herein warrant a next step toward in vivo studies, to confirm the potential use of HA-L-PNIPAM 0.5 as a viscosupplement treatment for osteoarthritis. Thanks to the high versatility of characteristics and applications, the thermosensitivity of the HA-L-PNIPAM derivatives could be used for many alternative applications. For instance, in aesthetic surgery to facilitate the injection of volume fillers, or as in situ forming implant to treat stress urinary incontinence by endoscopic submucosal injections or as a vehicle for cell therapy. Application as novel drug delivery vehicles might also be envisioned to form drug depots in hard-to-reach pathological sites.

## 5. Patents

The polymers developed in this study fall in the field of US Patent US 10,767,037 B2.

## Figures and Tables

**Table 1:** Summary of viscoelastic properties obtained in rheology for Ostenil^®^ and different HA-L-PNIPAM 0.5 concentrations at 25 °C and 37 °C. HA contents for Ostenil^®^ were calculated according to values obtained in ^1^H NMR spectroscopy.

Viscosupplement	HA Content (mg/mL)	Temperature (°C)	Frequency: 0.5 Hz	
			G′ (Pa)	G″ (Pa)
Ostenil^®^	10.0	25	8.24 ± 0.60	13.01 ± 0.75
		37	5.35 ± 0.46	10.30 ± 0.64
HA-L-PNIPAM 0.5 7%	9.9	25	2.12 ± 2.27	3.37 ± 1.97
		37	150.39 ± 31.90	62.60 ± 15.88
HA-L-PNIPAM 0.5 5%	7.1	25	0.01 ± 0.01	0.22 ± 0.01
		37	29.11 ± 3.01	11.97 ± 3.10
HA-L-PNIPAM 0.5 1%	1.4	25	0.02 ± 0.01	0.03 ± 0.01
		37	0.38 ± 0.15	0.27 ± 0.02

**Table 2:** Summary of HA-L-PNIPAM 0.5 5% contents calculated according to values obtained in ^1^H NMR spectroscopy.

Viscosupplement	HA Content (mg/mL)	Sulfo DBCO-PEG4-NH_2_ Content (mg/mL)	PNIPAM-N_3_ Content (mg/mL)
HA-L-PNIPAM 0.5 5%	7.1	2.1	40.8

## Data Availability

The data presented in this study are available on request from the corresponding author.

## Supplementary Materials

The following supporting information can be downloaded at https://www.mdpi.com/article/10.3390/pharmaceutics14030659/s1, Figure S1: ^1^H-NMR spectra in D_2_O of HA (A), HA-Sulfo DBCO-PEG4 with the DS_1_ (B), and HA-L-PNIPAM 0.5 with the DS_2_ (C). For B and C, the polymers were submitted to hyaluronidase before NMR (see methods). The specific assignments of the proton NMR resonance peaks are δ = 2.0 ppm (1), 4.7–2.7 ppm (HA-backbone/PEG-DBCO), 6.8–7.7 ppm (2), 1.1 ppm (3). DS1 (HA-Sulfo DBCO-PEG4) = 16.9%. DS2 (HA-L-PNIPAM 0.5) = 86,3%; Figure S2: ^1^H-NMR spectra in D_2_O of HA (A), HA-Sulfo DBCO-PEG4 with the DS_1_ (B), and HA-L-PNIPAM 0.5 with the DS_2_ (C). For B and C, the polymers were submitted to hyaluronidase before NMR (see methods). The specific assignments of the proton NMR resonance peaks are δ = 2.0 ppm (1), 4.7–2.7 ppm (HA-backbone/PEG-DBCO), 6.8–7.7 ppm (2), 1.1 ppm (3). DS_1_ (HA-Sulfo DBCO-PEG4) = 6.25%. DS2 (HA-L-PNIPAM 0.5) = 87,5%; Figure S3: ^1^H-NMR spectra in D_2_O of HA (A), HA-Sulfo DBCO-PEG4 0.1 (B), and HA-L-PNIPAM 0.1 (C). For B and C, the polymers were submitted to hyaluronidase before NMR (see methods). The specific assignments of the proton NMR resonance peaks are δ = 2.0 ppm (1), 4.7–2.7 ppm (HA-backbone/PEG-DBCO), 6.8–7.7 ppm (2), 1.1 ppm (3). DS (HA-Sulfo DBCO-PEG4) = 1.125%. DS (HA-L-PNIPAM 0.1) = 49.5%; Figure S4: ^1^H-NMR spectra in D_2_O of HA (A) and HA-PNIPAM (B). The specific assignments of the proton NMR resonance peaks are δ = 2.0 ppm (1) (i.e., acetamido moiety of the N-acetyl-D-glucosamine residue of HA), 1.1 ppm (2) (i.e., methyl protons of PNIPAM); Figure S5: ATR/FT-IR spectra recorded on HA (blue curve), HA-Sulfo DBCO-PEG4 (orange curve) and HA-L-PNIPAM 0.5 (black curve); Figure S6: ATR/FT-IR spectra recorded on HA (blue curve) and HA-PNIPAM (black curve); Table S1: Degrees of substitution (DS) of the intermediate HA-Sulfo DBCO-PEG4 (DS_1_) for the formulations of HA-L-PNIPAM determined by UV-vis spectroscopy at 307 nm; Table S2: Degrees of substitution (DS) of HA-PNIPAM determined by UV-vis spectroscopy at 286 nm; Figure S7: Temperature dependence of the storage modulus (G′) and loss modulus (G″) at a frequency of 2.5 Hz of Ostenil^®^, HA-L-PNIPAM 0.5 1%, 5%, and 7% (*n* = 3; ±sd) (A and B); Table S3: Summary of viscoelastic properties obtained in rheology for Ostenil^®^ and different HA-L-PNIPAM 0.5 concentrations at 25 °C and 37°C. HA contents for Ostenil^®^ were calculated according to values obtained in ^1^H NMR spectroscopy; Figure S8: Ostenil^®^ and HA-L-PNIPAM 0.5 5% initial storage modulus (G′) and initial loss modulus (G″), after 28 days of storage, at 0.5 Hz and 37°C and after 4 additions of 10 µL of hyaluronidase (50 U/mL) (A and B). Ostenil^®^ and HA-L-PNIPAM 0.5 5% initial storage modulus (G′) and initial loss modulus (G″), as function of time at 0.5 Hz and 37°C, after an addition of 40 µL H_2_O_2_ (30% *w/w*) (E and F) and after 6 h (C and D). Significant difference (i.e., *p* < 0.05) was evidenced by one asterisk (“*”). Highly statistically significative differences (i.e., *p* < 0.0001) was evidenced by four asterisks (“****”). *n* = 3 ± SD; Figure S9: Viscosity of HA-L-PNIPAM 0.5 5% under a constant shear stress of 0.1 s^−1^ at 37 °C before and after 0.22 µm filtration. Highly statistically significative differences (i.e., *p* < 0.0001) was evidenced by four asterisks (“****”). *n* = 3 ± SD; Figure S10: The cellular viability of human fibroblast like synoviocytes (HFLS) incubated with HA-L-PNIPAM 0.5 formulations at 1%, 5% and 7% (*w/v*) after 24 h. Significant difference (i.e., *p* < 0.05) was evidenced by one asterisk (“*”). Highly statistically significative differences (i.e., *p* < 0.001) was evidenced by three asterisks (“***”). *n* = 4 ± SD; Table S4: Results of viability obtained by Countess^®^ after 0.4% trypan blue staining of human fibroblast-like synoviocytes (HFLS) incubated with HA-L-PNIPAM 0.5 5%, HA-L-PNIPAM 0.5 5% digested by hyaluronidase, Hyaluronic acid 0,71% (2200–2400 kDa), Sulfo DBCO-PEG4-NH_2_ 0.21%, PNIPAM-N_3_ 4.08% (15′000 Da) and Ostenil^®^ after 24 h and 72 h. (*n* = 3; ±sd); Figure S11: Aspects of the cells after 24 h and 72 h of incubation with the different products; Figure S12: HA-L-PNIPAM 0.5 5% storage and loss modulus after 30 days of storage at 4 °C. Highly statistically significative differences (i.e., *p* < 0.0001) was evidenced by four asterisks (“****”). *n* = 3 ± SD. Figure S13: Summary of possible future application for HA-L-PNIPAM.

## Abbreviations

Da	Daltons
DLS	Dynamic light scattering
DS	Degree of substitution
EDC	*N*-(3-dimethylaminopropyl)-*N*′-ethylcarbodiimide
G′	The storage/elastic moduli
G″	The loss/viscous moduli
HA	Hyaluronic acid
HFLS	Human Fibroblast-like Synoviocytes
HMW	High molecular weight
H_2_O_2_	Hydrogen peroxide
LCST	Lower critical solution temperature
LVE	Linear viscoelastic region
MW	Molecular weight
NHS	*N*-hydroxysuccinimide
Pa	Pascals
Pa·s	Pascal seconds
PBS	Phosphate-buffered saline
OA	Osteoarthritis
PNIPAM	poly(*N*-isopropylacrylamide)
ROS	Reactive oxygen species
SEM	Scanning electron microscopy
