# Supplementary material for: Nanoforming Hyaluronan-Based Thermoresponsive Hydrogels: Optimized and Tunable Functionality in Osteoarthritis Management
Source: Pharmaceutics. 2022 Mar 17;14(3):659. doi: 10.3390/pharmaceutics14030659 (PMC8955188; doi:10.3390/pharmaceutics14030659)
Supplement: Supplementary file 1 [file pharmaceutics-14-00659-s001.zip › pharmaceutics-1619705-supplementary.pdf]

pharmaceutics

MDPI

Supplementary Materials: Nanoforming Hyaluronan-Based Thermoresponsive Hydrogels: Optimized and Tunable Functionality in Osteoarthritis Management

Alexandre Porcello, Paula Gonzalez-Fernandez, Olivier Jordan and Eric Allémann

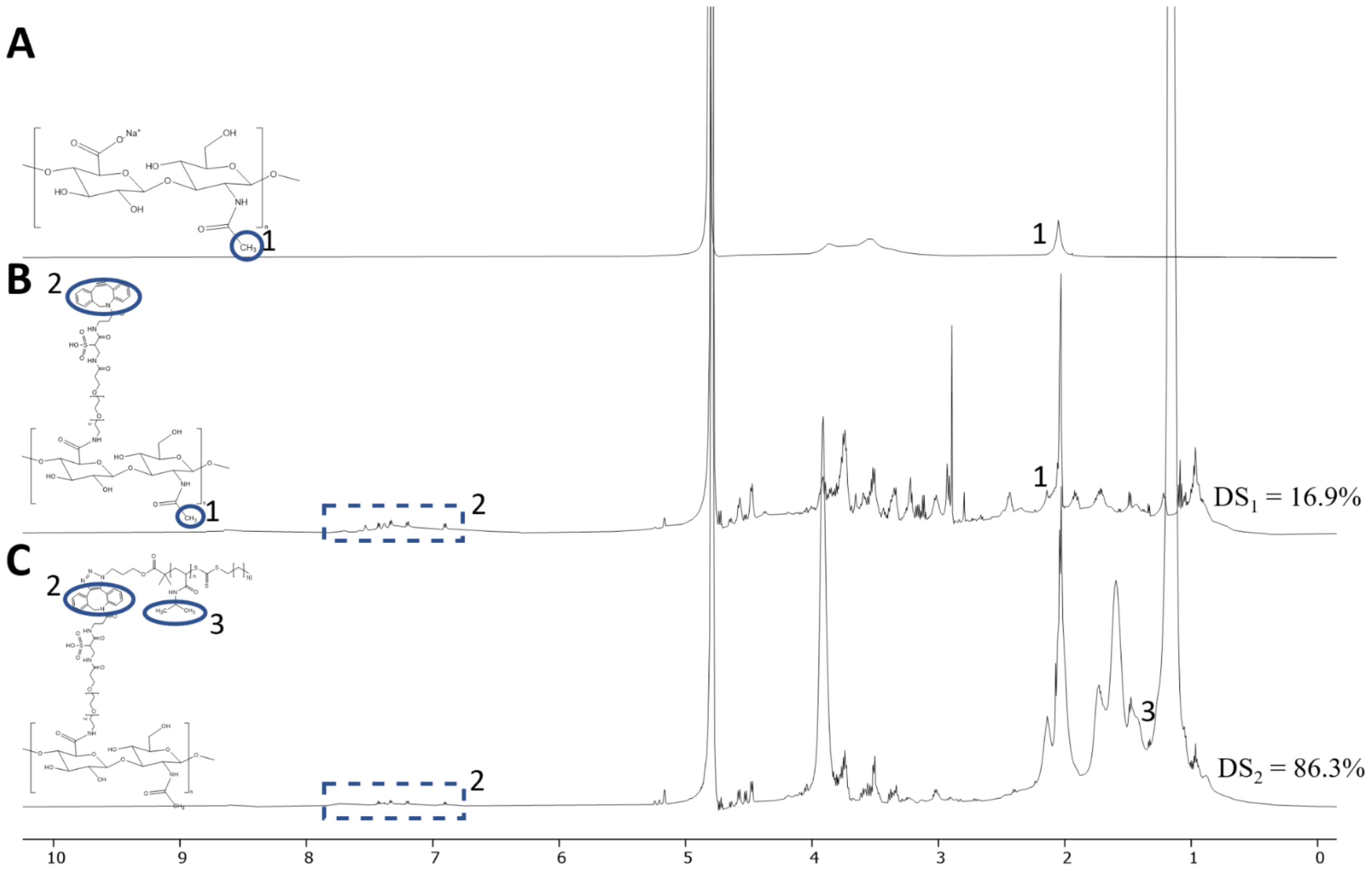

Figure S1. 1H-NMR spectra in D2O of HA (A), HA - Sulfo DBCO-PEG4 with the DS1 (B), and HA-L-PNIPAM 0.5 with the DS2 (C). For B and C, the polymers were submitted to hyaluronidase before NMR (see methods). The specific assignments of the proton NMR resonance peaks are δ = 2.0 ppm (1), 4.7–2.7 ppm (HA-backbone/PEG-DBCO), 6.8–7.7 ppm (2), 1.1 ppm (3). DS1 (HA - Sulfo DBCO-PEG4) = 16.9%. DS2 (HA-L-PNIPAM 0.5) = 86.3%.

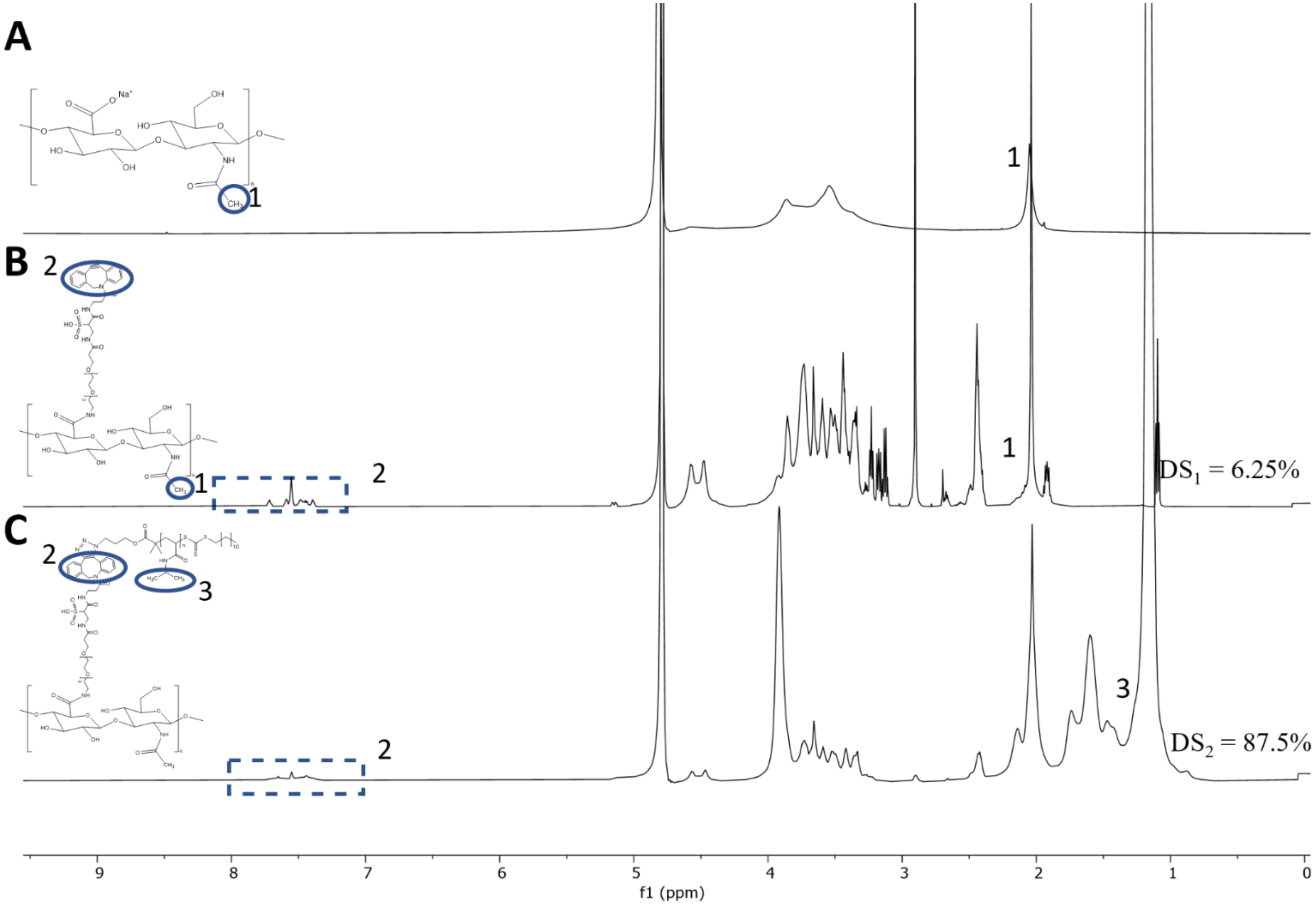

Figure S2. 1H-NMR spectra in D2O of HA (A), HA - Sulfo DBCO-PEG4 with the DS1 (B), and HA-L-PNIPAM 0.5 with the DS2 (C). For B and C, the polymers were submitted to hyaluronidase before NMR (see methods). The specific assignments of the proton NMR resonance peaks are δ = 2.0 ppm (1), 4.7–2.7 ppm (HA-backbone/PEG-DBCO), 6.8–7.7 ppm (2), 1.1 ppm (3). DS1 (HA - Sulfo DBCO-PEG4) = 6.25%. DS2 (HA-L-PNIPAM 0.5) = 87.5%.

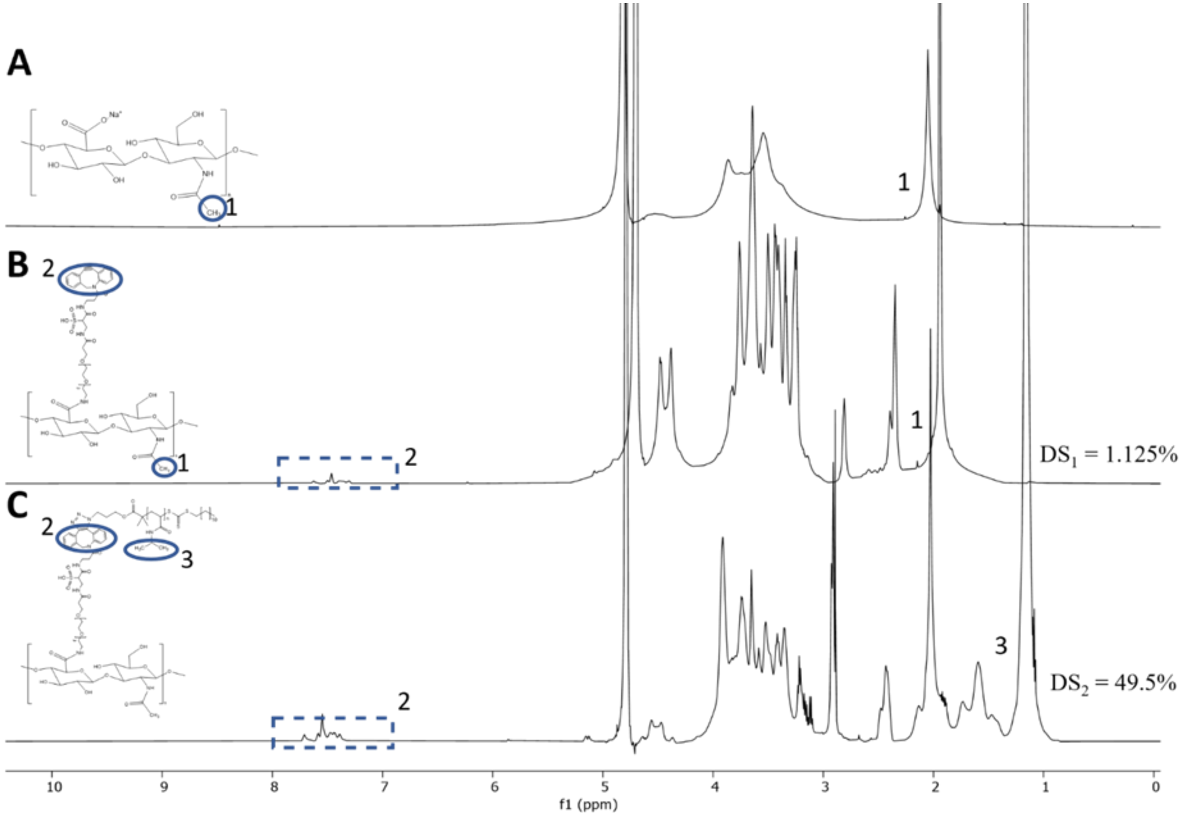

Figure S3. 1H-NMR spectra in D2O of HA (A) HA - Sulfo DBCO-PEG4 0.1 (B) and HA-L-PNIPAM 0.1 (C). For B and C, the polymers were submitted to hyaluronidase before NMR (see methods). The specific assignments of the proton NMR resonance peaks are δ = 2.0 ppm (1), 4.7–2.7 ppm (HA-backbone/PEG-DBCO), 6.8–7.7 ppm (2), 1.1 ppm (3). DS (HA - Sulfo DBCO-PEG4) = 1.125%. DS (HA-L-PNIPAM 0.1) = 49.5%.

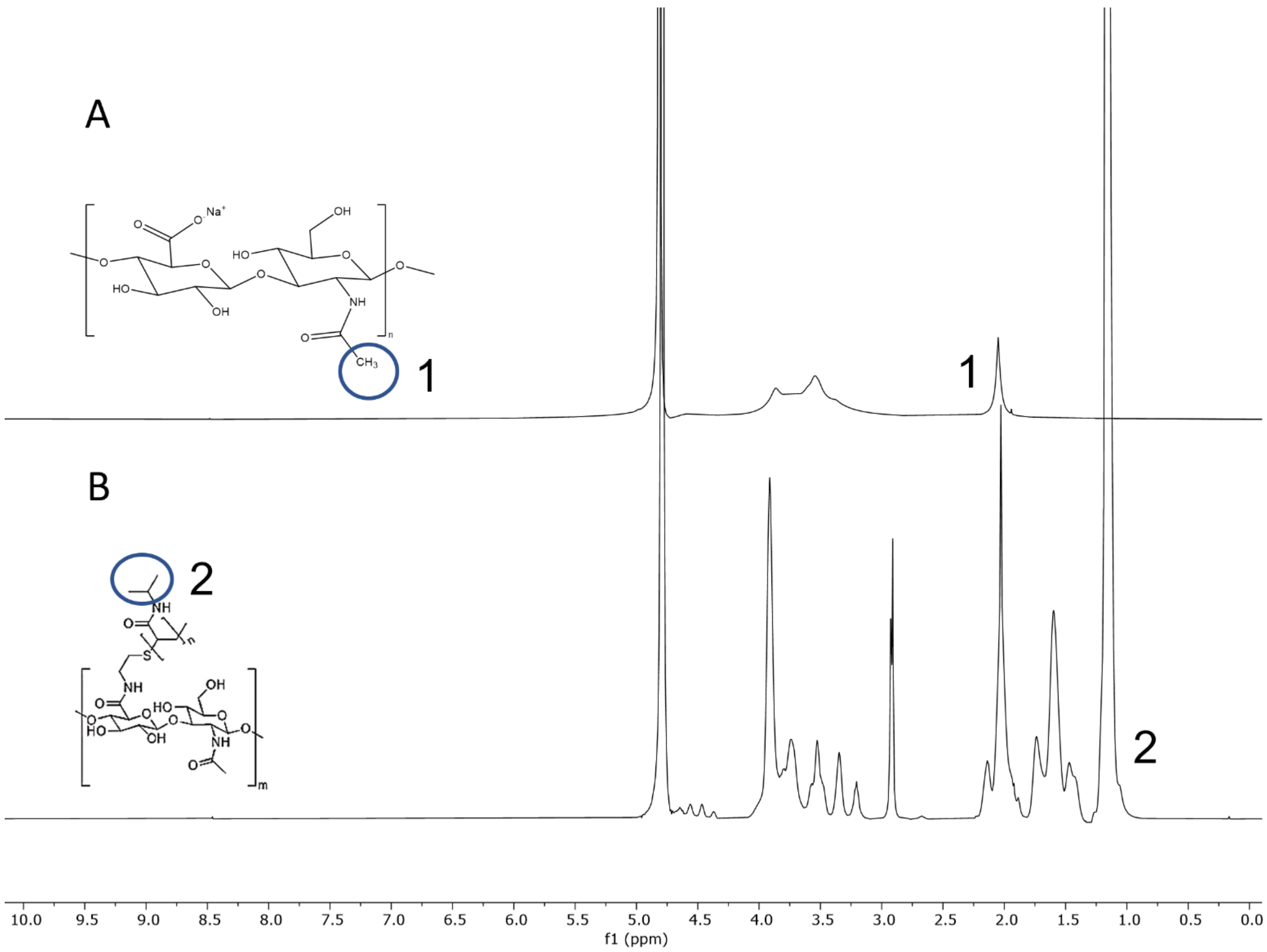

Figure S4. 1H-NMR spectra in D2O of HA (A) and HA - PNIPAM (B). The specific assignments of the proton NMR resonance peaks are δ = 2.0 ppm (1) (i.e., acetamido moiety of the N-acetyl-D-glucosamine residue of HA), 1.1 ppm (2) (i.e., methyl protons of PNIPAM).

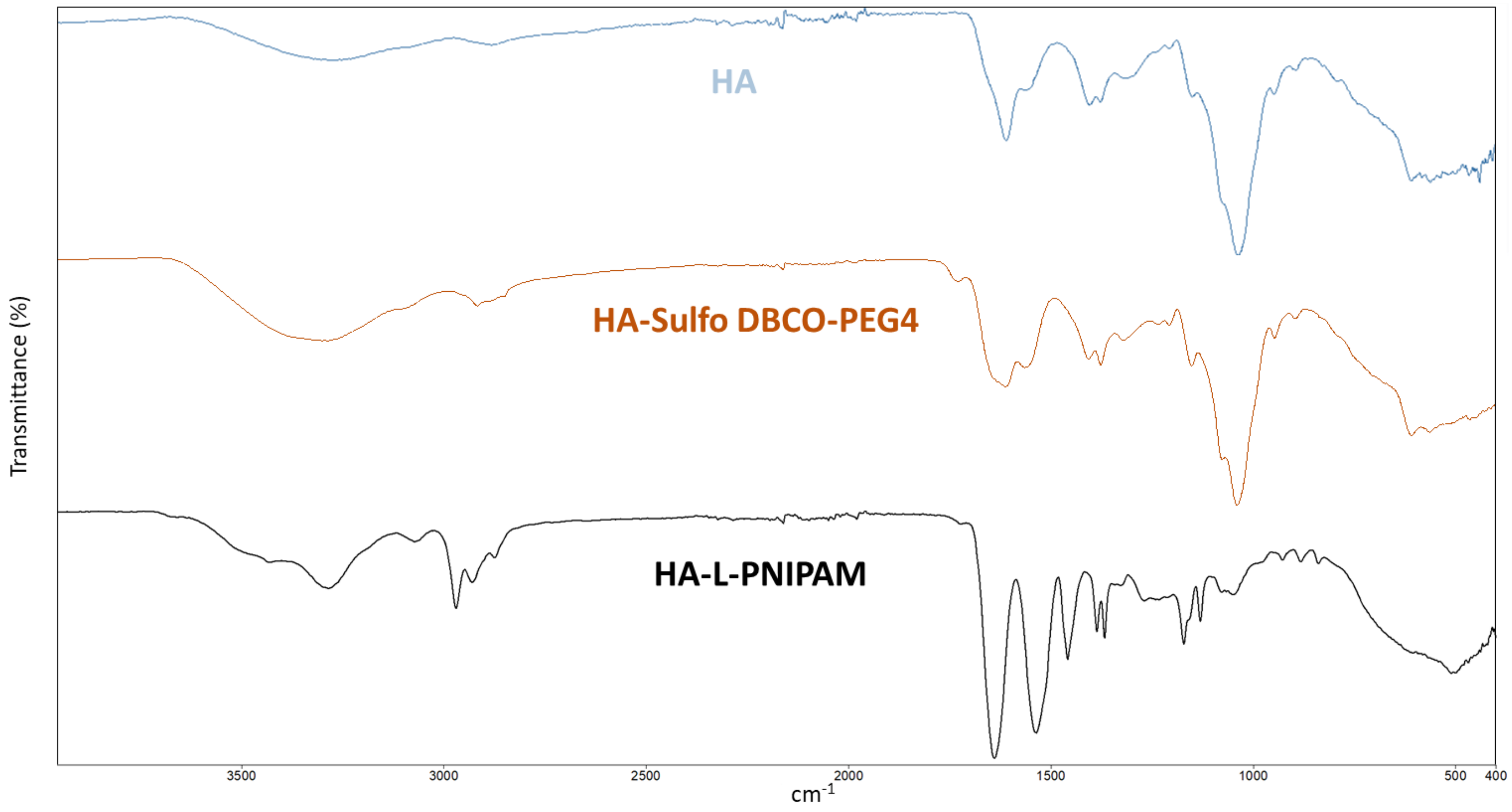

Figure S5. ATR/FT-IR spectra recorded on HA (blue curve), HA-Sulfo DBCO-PEG4 (orange curve) and HA-L-PNIPAM 0.5 (black curve).

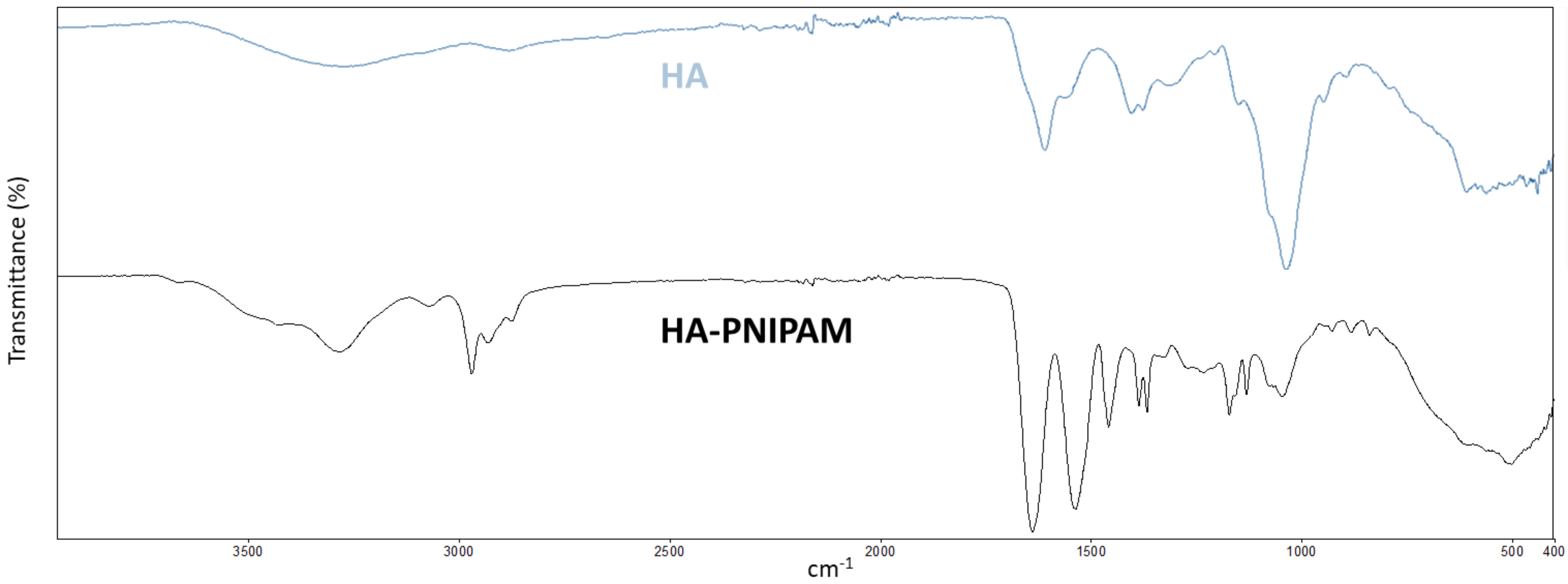

Figure S6. ATR/FT-IR spectra recorded on HA (blue curve) and HA-PNIPAM (black curve).

Table S1. Degrees of substitution (DS) of the intermediate HA - Sulfo DBCO-PEG4 (DS1) for the formulations of HA-L-PNIPAM determined by UV-vis spectroscopy at 307 nm.

**Formulation**	**DS_1 (307 nm)**
HA-L-PNIPAM 0.5	13.8
HA-L-PNIPAM 0.25	6.2
HA-L-PNIPAM 0.1	0.9

Table S2. Degrees of substitution (DS) of HA-PNIPAM determined by UV-vis spectroscopy at 286 nm.

**Formulation**	**DS (286 nm)**
HA-PNIPAM	8.0

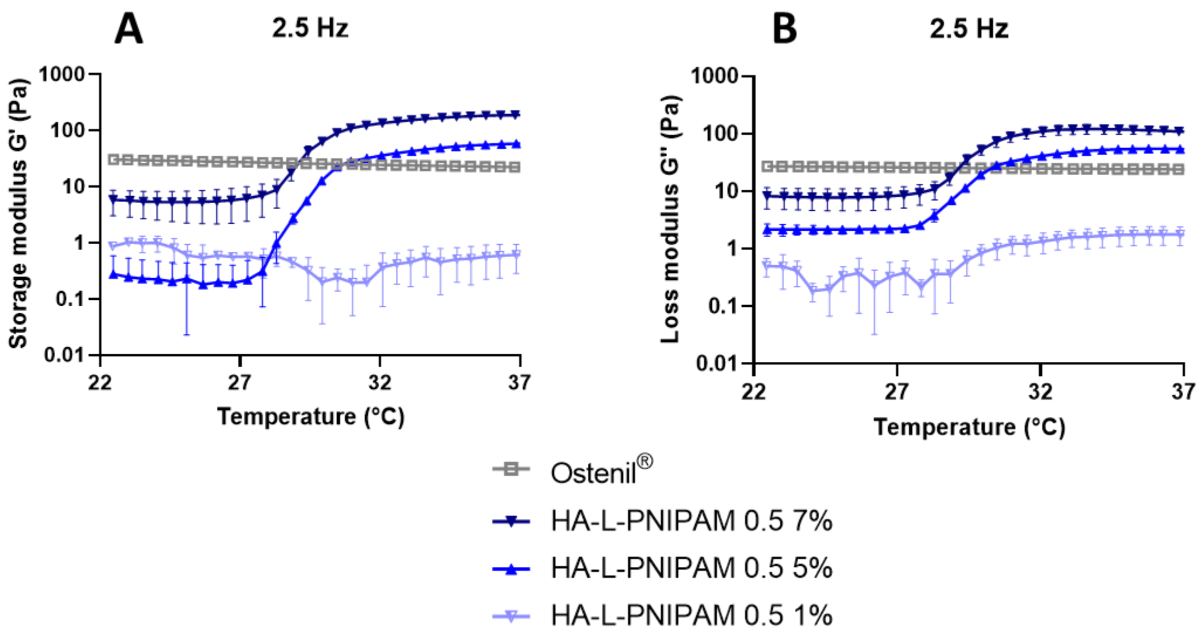

Figure S7. Temperature dependence of the storage modulus (G′) and loss modulus (G″) at a frequency of 2.5 Hz of Ostenil®, HA-L-PNIPAM 0.5 1 %, 5 %, and 7 % (n = 3; ± SD) (A and B).

Table S3. Summary of viscoelastic properties obtained in rheology for Ostenil® and different HA-L-PNIPAM 0.5 concentrations at 25 °C and 37°C. HA contents for Ostenil® were calculated according to values obtained in ^{1}H NMR spectroscopy.

Viscosupplement	HA content (mg/mL)	Temperature (°C)	Frequency 2.5 Hz	
			G' (Pa)	G' (Pa)
Ostenil®	10.0	25	28.52 ± 1.1	28.52 ± 1.1
		37	22.1 ± 0.67	22.1 ± 0.67
HA-L-PNIPAM 0.5 7%	9.9	25	5.28 ± 3.03	5.28 ± 3.03
		37	189.51 ± 21.31	189.51 ± 21.31
HA-L-PNIPAM 0.5 5%	7.1	25	0.23 ± 0.21	0.23 ± 0.21
		37	59.23 ± 5.97	59.23 ± 5.97
HA-L-PNIPAM 0.5 1%	1.4	25	0.68 ± 0.27	0.68 ± 0.27
		37	0.63 ± 0.33	0.63 ± 0.33

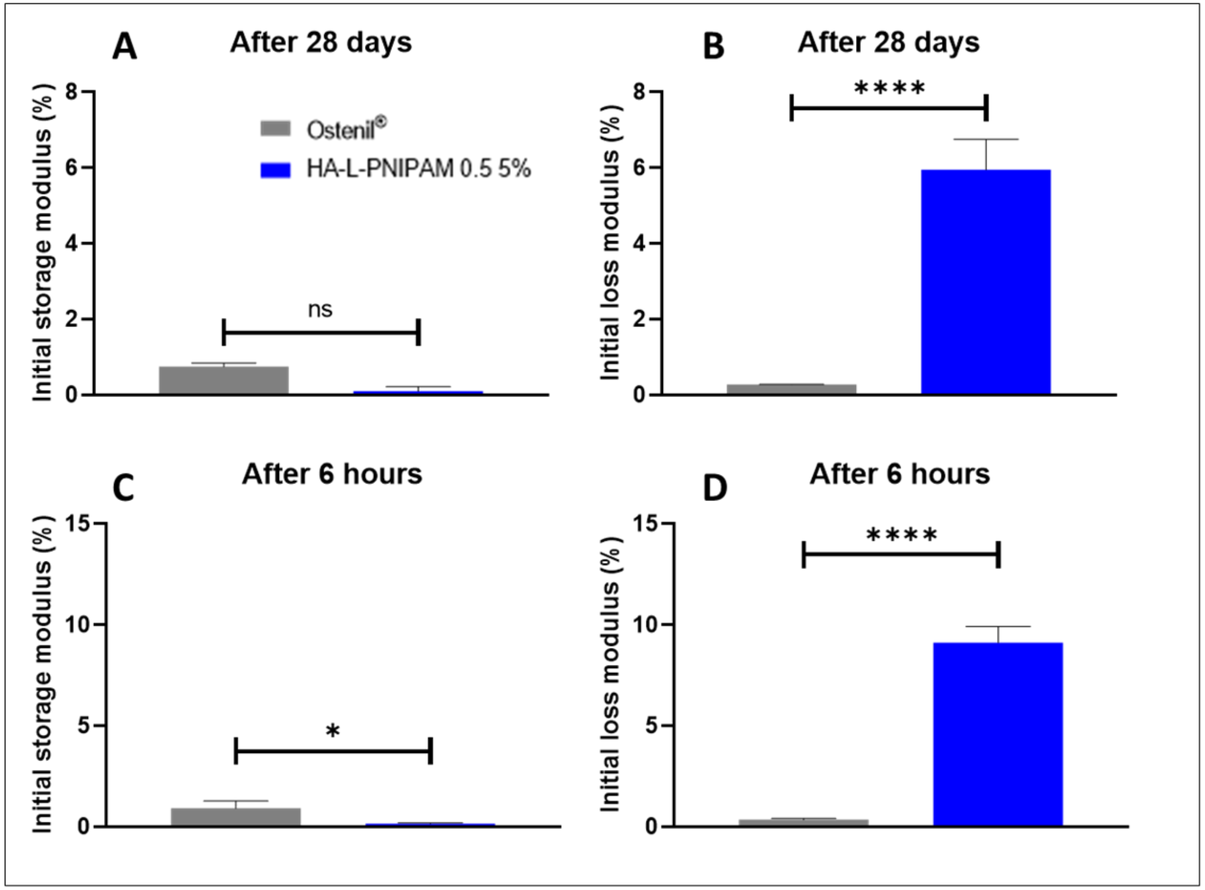

Figure S8. Ostenil® and HA-L-PNIPAM 0.5 5 % initial storage modulus (G′) and initial loss modulus (G″), after 28 days of storage, at 0.5 Hz and 37°C and after 4 additions of 10 µl of hyaluronidase (50 U/mL) (A and B). Ostenil® and HA-L-PNIPAM 0.5 5% initial storage modulus (G′) and initial loss modulus (G″), as function of time at 0.5 Hz and 37°C, after an addition of 40 µl H_2O_2 (30% w/w) (E and F) and after 6 hours (C and D). Significant difference (i.e., $p < 0.05$) was evidenced by one asterisk ("*"). Highly statistically significative differences (i.e., $p < 0.0001$) was evidenced by four asterisks ("****"). n = 3 ± SD

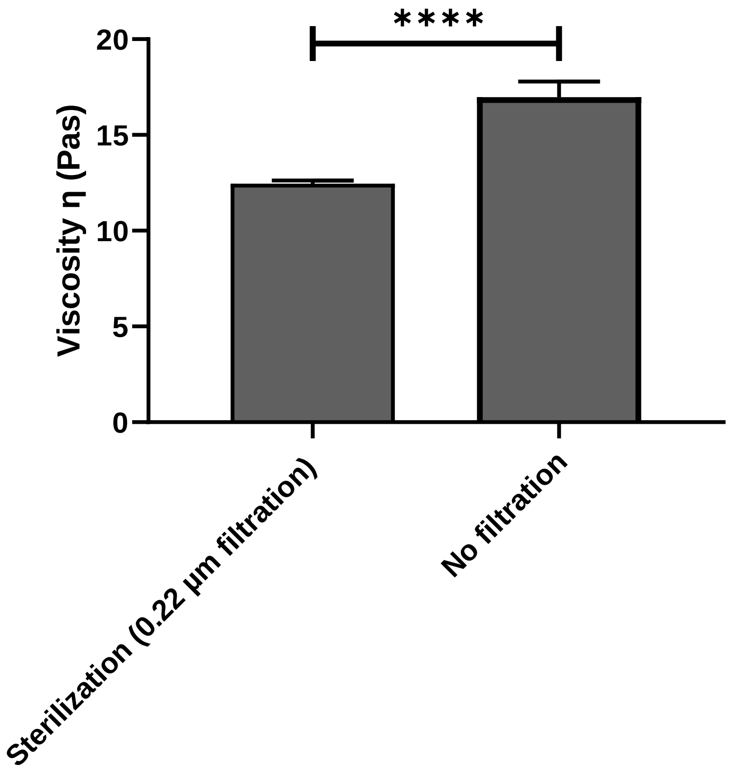

Figure S9. Viscosity of HA-L-PNIPAM 0.5 5% under a constant shear stress of 0.1 s^{-1} at 37 °C before and after 0.22 µm filtration. Highly statistically significative differences (i.e., $p < 0.0001$) was evidenced by four asterisks ("****"). n = 3 ± SD

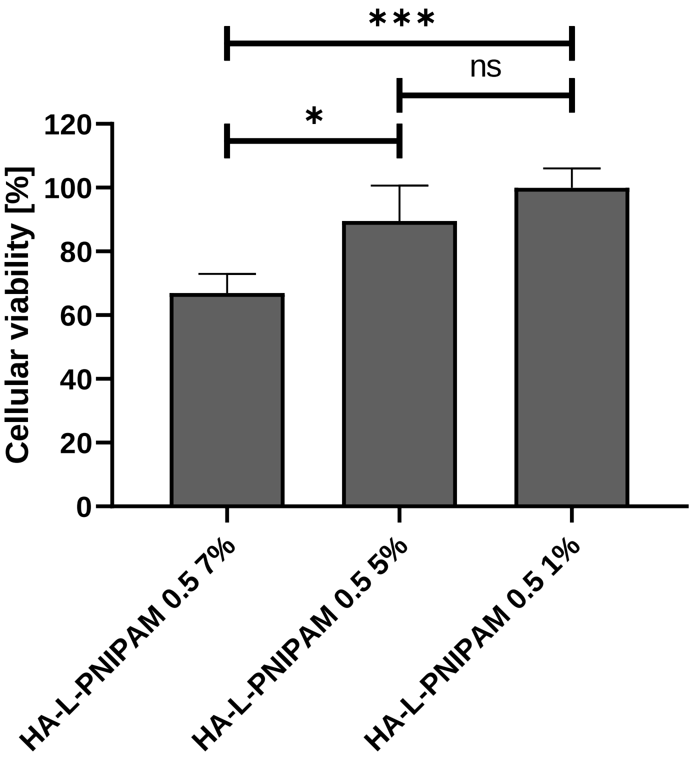

Figure S10. The cellular viability of human fibroblast like synoviocytes (HFLS) incubated with HA-L-PNIPAM 0.5 formulations at 1 %, 5 % and 7 % (w/v) after 24 h. Significant difference (i.e., $p < 0.05$) was evidenced by one asterisk ("*"). Highly statistically significative differences (i.e., $p < 0.001$) was evidenced by three asterisks ("***"). n = 4 ± SD

Table S4. Results of viability obtained by Countess® after 0.4% trypan blue staining of human fibroblast-like synoviocytes (HFLS) incubated with HA-L-PNIPAM 0.5 5%, HA-L-PNIPAM 0.5 5% digested by hyaluronidase, Hyaluronic acid 0,71 % (2200–2400 kDa), Sulfo DBCO-PEG4-NH_2 0.21 %, PNIPAM-N_3 4.08 % (15’000 Da) and Ostenil® after 24 h and 72 h. (n = 3; ± SD).

Compounds	Viability	
	**24 hours**	**72 hours**
Media	100 ± 0	99.3 ± 0.5
HA-L-PNIPAM 0.5	98.7 ± 1.2	93.6 ± 9.3
HA	99.3 ± 1.2	98.7 ± 1.2
Sulfo DBCO-PEG4-NH_2	98.7 ± 1.2	90 ± 13
PNIPAM-N_3	96.0 ± 5.3	78 ± 16.0
HA-L-PNIPAM 0.5 Digested	96.0 ± 2.6	81 ± 18.2
Ostenil®	96.6.23 ± 4.1	85 ± 6.1

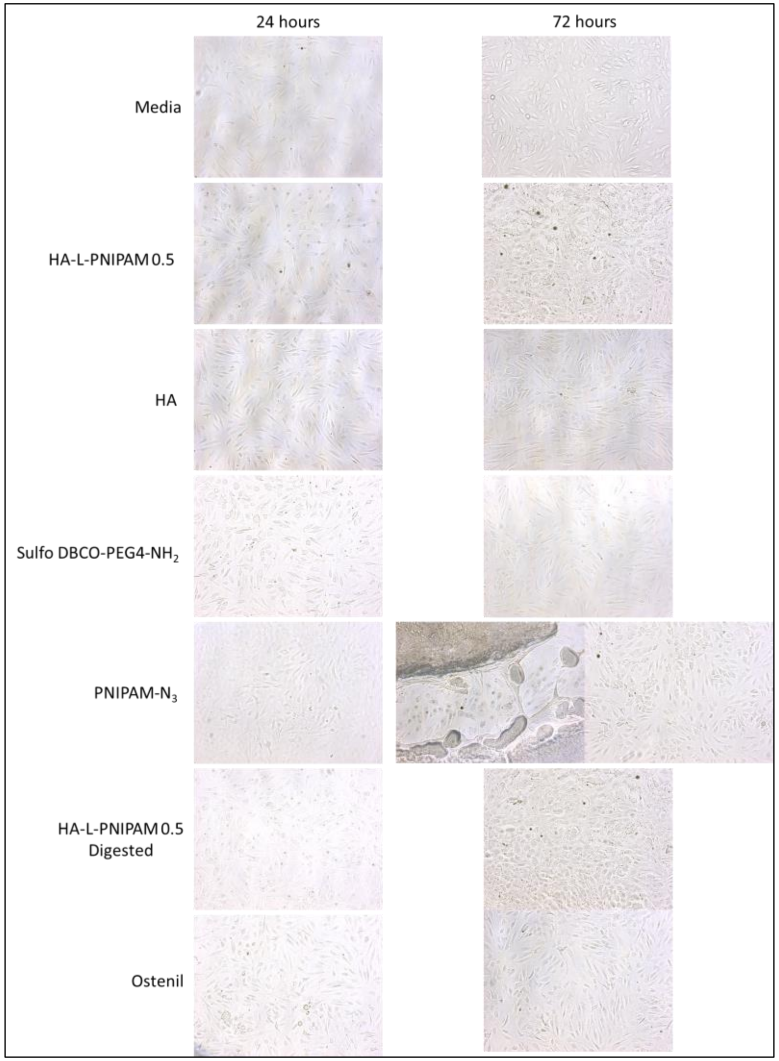

Figure S11. Aspects of the cells after 24 hours and 72 hours of incubation with the different products.

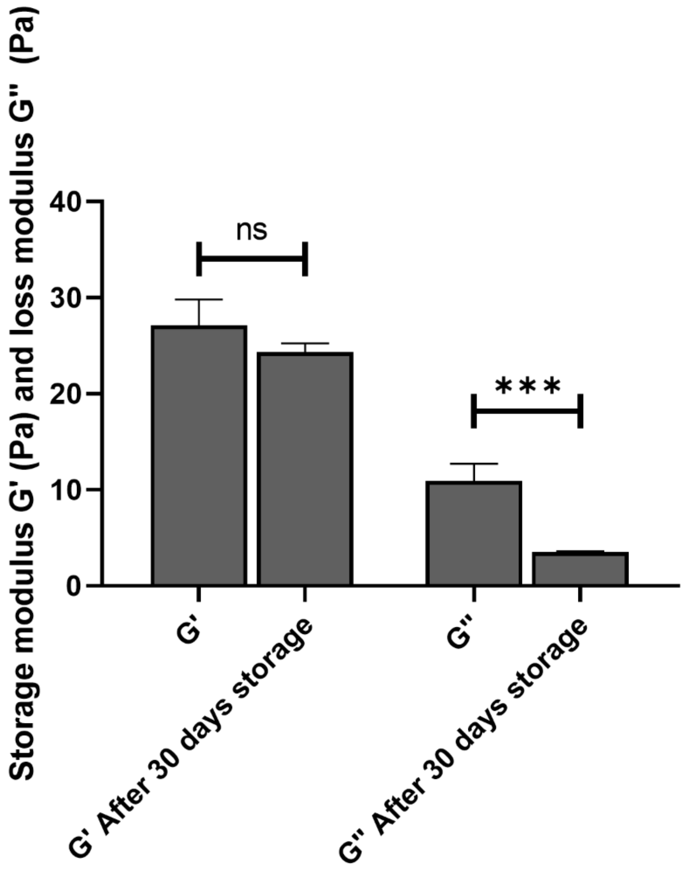

Figure S12. HA-L-PNIPAM 0.5 5% storage and loss modulus after 30 days of storage at 4 °C. Highly statistically significative differences (i.e., $p < 0.001$) was evidenced by four asterisks ("***"). n = 3 ± SD.

	Musculoskeletal indications	Drug delivery system	Aesthetic indications	Therapeutic cell delivery	Other applications
	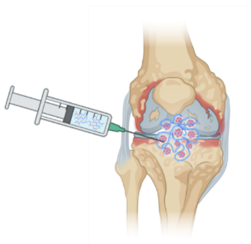	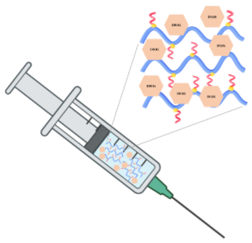	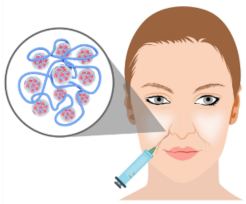	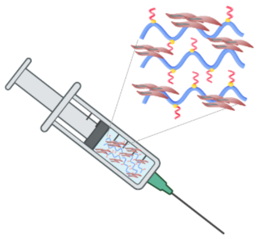	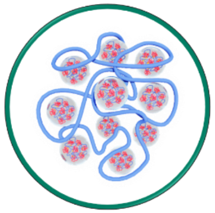
Identified specific applications	• Viscosupplementation<br>• Volumetric supplementation<br>• Tendinous tissue disorders	• Small drug<br>• Protein<br>• Peptide	• Facial dermal filler<br>• Body filler<br>• Cosmetics	• Living cells<br>• Cellular derivatives	• Stress urinary incontinence
Main advantages	✓ Resistance against hyaluronidases<br>✓ Resistance against ROS<br>✓ High *in situ* viscosity	✓ Injectability<br>✓ *In situ* forming implant	✓ Ease of injection<br>✓ Thinner needle<br>✓ Resistance against hyaluronidases<br>✓ Resistance against ROS<br>✓ High *in situ* viscosity	✓ Ease of injection<br>✓ Viability after extrusion	✓ Ease of injection<br>✓ *In situ* forming implant

Figure S13. Summary of possible future application for HA-L-PNIPAM.
